# Impact of glucose and lipid metabolic disorders on the risk of chronic post-operative pain syndrome after colorectal cancer surgery

**DOI:** 10.3389/fonc.2025.1633208

**Published:** 2025-09-18

**Authors:** Shuaichao Li, Longxin Fan, Tao Meng, Zhengjie Gao

**Affiliations:** Department of General Surgery, The First Affiliated Hospital of Xinxiang Medical University, Weihui/Xinxiang, Henan, China

**Keywords:** anxiety, chronic pain, colorectal cancer, depression, metabolism, quality of life

## Abstract

**Background:**

The influencing factors of post-operative chronic pain syndrome after colorectal cancer surgery (POCPS-CCCS) remain unclear. This study aimed to explore the impact of glucose and lipid metabolic disorders on the risk of this postoperative complication.

**Methods:**

Case-control study: At 3 months after surgery (defined as baseline), 513 colorectal cancer patients were divided into chronic pain group and control group based on their POCPS-CCCS status. Multivariate logistic or linear regression was used to analyze the associations of glucose and lipid metabolic disorders and peripheral inflammation (assessed via IL-1β, IL-6, and TNF-α) with the risk of POCPS-CCCS. Cohort study: Among the 513 patients, 352 without POCPS-CCCS at baseline were further divided into metabolic disorder subgroup and metabolic normal subgroup according to their baseline glucose and lipid metabolic status. Over a 9-month follow-up (from 4 to 12 months after surgery), multivariate logistic or linear regression was used to investigate the impacts of the metabolic disorders and peripheral inflammation at baseline on the risk of POCPS-CCCS during the follow-up. In both study components, the associations of the metabolic disorders, peripheral inflammation, and POCPS-CCCS with patients’ depression, anxiety, and quality of life scores were also evaluated.

**Results:**

Combined findings from the case-control and cohort studies showed that: At baseline, glucose and lipid metabolic disorders were significantly associated with elevated peripheral inflammation (all P<0.05). The metabolic disorders and elevated peripheral inflammation at baseline were associated with an increased risk of POCPS-CCCS during the follow-up (all P<0.05). Furthermore, the baseline metabolic disorders, elevated baseline peripheral inflammation, and incident POCPS-CCCS during the follow-up were associated with worse depression, anxiety, and lower quality of life scores at the follow-up end (all P<0.05).

**Conclusion:**

These results preliminarily indicate that glucose and lipid metabolic disorders, together with peripheral inflammation, may promote the development of POCPS-CCCS. Additionally, these metabolic and inflammatory disorders, alongside POCPS-CCCS, may further impair patients’ psychological status and quality of life. Thus, improving these pathophysiological abnormalities and reducing the risk of this postoperative complication may contribute to health maintenance in patients after colorectal cancer surgery.

## 1 Introduction

Post-operative chronic pain syndrome after colorectal cancer surgery (POCPS-CCCS) is defined as persistent or recurrent pain in the surgical area that lasts for ≥3 months after colorectal cancer surgery, excluding pain caused by tumor recurrence, metastasis, or other unrelated etiologies (1, 2). Epidemiological data indicate that its incidence varies widely (3%-50%) due to differences in assessment scales, surgical techniques, and individual patient characteristics (3–5). The clinical impact of POCPS-CCCS is substantial: persistent pain induces long-term stress, increasing the risk of anxiety and depression, while disrupting sleep, appetite, and daily activities, thereby significantly reducing patients’ quality of life.

The pathogenesis of POCPS-CCCS involves multiple potential mechanisms and influencing factors (3–6). Surgically, direct tissue and nerve damage can trigger abnormal nerve fiber growth and altered conduction pathways, leading to persistent pain signaling. Postoperative inflammatory responses activate peripheral nerve pain receptors, enhancing pain sensitivity, and varying surgical trauma may further modulate chronic pain incidence. Patient-specific factors — such as age, gender, comorbidities, and psychological status — also play non-negligible roles, as they influence nerve repair and pain perception. However, the precise mechanisms and key drivers of POCPS-CCCS remain poorly understood.

Although conclusive evidence is lacking, theoretical frameworks suggest that glucose and lipid metabolic disorders may contribute to the development of POCPS-CCCS. From the perspective of glucose metabolism, hyperglycemia can inhibit the mitochondrial deacetylase Sirt3, thereby activating aerobic glycolysis in microglia and promoting the release of inflammatory factors such as IL-1β to exacerbate neuroinflammation (7); it can also activate the TRPV4 channel in dorsal root ganglia, triggering neuronal oxidative stress and apoptosis, and inducing neuropathic pain (8). Additionally, diabetic patients have a significantly increased risk of postoperative opioid use, indicating that glucose metabolic disorders may elevate the risk of pain chronicity (9). From the perspective of lipid metabolism, obesity can increase oxidative stress and inflammation levels through the AMPK-ERK-NOX4 pathway, worsening neuropathic pain (10); obese patients exhibit higher postoperative pain scores and an increased risk of complications (11); moreover, lipid metabolic disorders disrupt the balance of lipid mediators in nerve tissue, impair the ability to resolve inflammation, and are unfavorable for pain relief (12). Notably, all these metabolic disorders are accompanied by the activation of inflammatory responses, which may collectively provide a pathological basis for the occurrence of POCPS-CCCS. Therefore, glucose and lipid metabolic disorders and their associated inflammatory responses may be involved in the pathogenesis of POCPS-CCCS, but this hypothesis requires empirical verification.

To address this knowledge gap, this study enrolled over 500 colorectal cancer surgery patients and employed a combined case-control and cohort design. It aimed to investigate the impacts of glucose and lipid metabolic disorders and elevated peripheral inflammation on POCPS-CCCS development, as well as their effects on patients’ psychological status and quality of life. These findings are expected to deepen understanding of POCPS-CCCS pathogenesis and inform targeted prevention and management strategies.

## 2 Materials and methods

### 2.1 Study design

**Figure 1:**
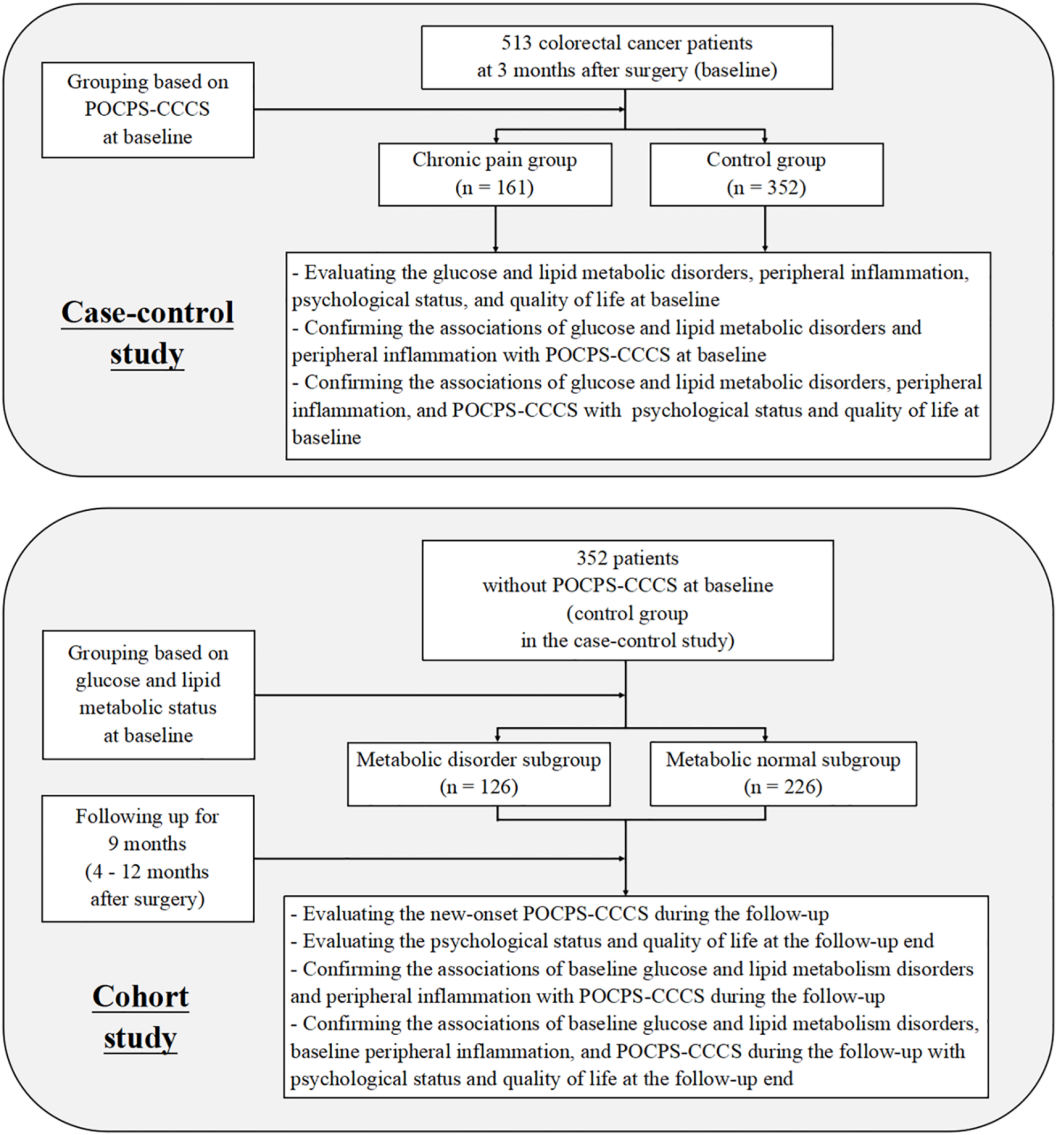
Flow diagram of the study. POCPS-CCCS, Post-operative chronic pain syndrome after colorectal cancer surgery.

This study adopted a nested design comprising two components: a case-control study and a cohort study. This dual design enabled mutual validation of results from both components (**Figure 1**).

In the case-control study, patients were grouped into a chronic pain group and a control group based on the presence of POCPS-CCCS at 3 months after surgery (defined as baseline). This component aimed to analyze cross-sectional associations between glucose and lipid metabolic disorders, peripheral inflammation, and POCPS-CCCS, as well as their associations with patients’ psychological status and quality of life.

In the cohort study, the patients from the case-control study’s control group (without baseline POCPS-CCCS) were further subdivided into a metabolic disorder subgroup and a metabolic normal subgroup based on their baseline glucose and lipid metabolic status. A 9-month prospective follow-up (from 4 to 12 months after surgery) was conducted to record new-onset POCPS-CCCS. This component investigated the impacts of baseline glucose and lipid metabolic disorders and peripheral inflammation on POCPS-CCCS risk during the follow-up, as well as their effects on patients’ psychological status and quality of life at the follow-up end.

Baseline was set at 3 months after surgery, a time when acute trauma-related pain typically subsides, allowing clear differentiation between acute and chronic pain — consistent with POCPS-CCCS diagnostic criteria (≥3 months of pain). The 9-month follow-up period covered the high-incidence phase of POCPS-CCCS. To eliminate confounding by tumor recurrence, all participants were colorectal cancer patients without recurrence within 12 months after surgery, ensuring baseline data balance and accurate POCPS-CCCS assessment.

### 2.2 Participant inclusion at baseline

From January 2013 to June 2022, newly diagnosed colorectal cancer cases were recruited from 10 large communities in Xinxiang City, Henan Province, China; cases diagnosed before 2013 were excluded.

Recruitment procedures: Community doctors identified newly diagnosed patients during routine health record management. After obtaining informed consent, researchers contacted patients within weeks to collect data via telephone interviews and medical record reviews. Patients were enrolled if they met the following criteria (with baseline set at 3 months after surgery): (1) Pathologically confirmed colorectal cancer (13); (2) Underwent standardized tumor resection with complete removal of cancerous tissue; (3) Stable tumor status at 3 months after surgery; (4) Agreed to a 9-month follow-up (from 4 to 12 months after surgery) and cooperation with baseline and follow-up procedures (including medical record provision, interviews, and blood sampling); (5) No other severe diseases (e.g., other malignancies) throughout the study. Patients failing to meet these criteria were excluded.

### 2.3 Baseline assessment and data collection

First, two specialist physicians independently diagnosed POCPS-CCCS at baseline using refined criteria (1, 2, 14): (1) Postoperative pain persisted or recurred for ≥3 months; (2) Numerical rating scale (NRS) score ≥3 (0 = no pain, 10 = worst imaginable pain); (3) Pain localized to the surgical incision and surrounding areas (≤5 cm from the incision); (4) Pain characterized as stabbing, distending, or burning; (5) Serological tests (e.g., tumor markers) and imaging (e.g., abdominal CT, pelvic MRI) ruled out other etiologies (e.g., tumor recurrence, urinary calculi); (6) Special circumstances: Preoperative pain did not affect diagnosis; recurrent pain after acute pain resolution was diagnosed if duration and criteria were met; pain onset >12 months after surgery was excluded due to low surgical association.

Discrepancies between two specialist physicians were resolved via joint review, and inter-rater reliability for the first 100 patients (Cohen’s kappa = 0.83, 95% CI: 0.76–0.88, P<0.001) confirmed excellent consistency.

These diagnostic results of POCPS-CCCS were used to define the grouping of the case-control study (chronic pain vs. control groups).

Second, demographic, disease history, and tumor-related data were extracted from their medical records. Metabolic specialists assessed baseline glucose and lipid metabolic status using the following criteria: (1) Preoperative type 2 diabetes with documentation; (2) Oral glucose tolerance tests at baseline confirmed impaired fasting glucose (IFG) or impaired glucose tolerance (IGT) in patients with preoperative fasting glucose abnormalities (15); (3) Repeat fasting lipid profiling at baseline confirmed hypertriglyceridemia (HTG), hypercholesterolemia (HC), or high low-density lipoprotein cholesterol (HLDLC) in patients with preoperative lipid abnormalities. Serological testing for glucose and lipid metabolic disorders at baseline was conducted at the researchers’ affiliated hospital, while data on their preoperative status were obtained from their medical records. Patients having any one of the metabolic issues were classified as having glucose or lipid metabolic disorders, defining cohort subgroups (metabolic disorder vs. metabolic normal subgroups). Hypoglycemic/antilipemic treatment at baseline was recorded but not adjusted in models to reflect actual metabolic control.

Third, baseline peripheral IL-1β, IL-6, and TNF-α levels were measured at the researchers’ affiliated hospital using ELISA kits (ab214025, ab178013, ab181421, Abcam) (16). This timing minimized confounding by acute surgical stress or tumor-related inflammation, reflecting chronic inflammation relevant to postoperative complications.

Fourth, psychological status and quality of life were assessed using the Hospital Anxiety and Depression Scale (HADS) and Functional Living Index-Cancer (FLIC) (17, 18). HADS includes anxiety and depression subscales (scores: 0-21; higher scores indicate worse symptoms: 0-7 = normal, 8-10 = mild, 11-14 = moderate, 15-21 = severe). FLIC comprises 22 items (scores: 0-100; higher scores indicate better quality of life).

### 2.4 Follow-up

All baseline-enrolled patients underwent 9-month follow-up.

First, all patients underwent routine re-examinations (tumor markers, imaging) every 3–6 months to screen for recurrence, with 2–3 re-examinations completed during the follow-up. Researchers contacted patients around the time of re-examinations to confirm symptoms, ensure attendance, and review results. Patients with confirmed recurrence were excluded immediately; those lost to follow-up, refusing participation, or dying were also excluded.

Second, cohort study patients (metabolic disorder and metabolic normal subgroups) were contacted monthly via telephone to assess surgical area pain. Suspected POCPS-CCCS cases underwent home visits by two physicians for diagnosis using baseline criteria, and necessary examinations were completed at the researchers’ affiliated hospital (1, 2, 14).

Third, HADS and FLIC were re-administered to cohort patients at follow-up end to assess psychological status and quality of life (17, 18).

### 2.5 Data analysis

Two dedicated staff members managed data over the nearly 10-year study period, with immediate completeness checks and prompt follow-up for missing data.

Continuous variables were expressed as mean ± standard deviation, with group differences analyzed using independent-samples t-tests. Categorical variables were expressed as frequencies and percentages, with differences analyzed using chi-square tests.

Multivariate logistic or linear regression evaluated independent associations in both study components, adjusting for potential confounders: age, gender, nationality, essential hypertension, cardiovascular diseases, cerebrovascular diseases, tumor markers (i.e., carcinoembryonic antigen, cancer antigen 199, cancer antigen 242), tumor site, size, histological type, differentiation grade, stage, and postoperative treatment (i.e., adjuvant chemotherapy, targeted therapy, immunotherapy, traditional Chinese medicine therapy). Stepwise regression was not used to exclude weakly associated factors, ensuring comprehensive confounding control and method reproducibility. Hypoglycemic/antilipemic medication history was not adjusted to reflect actual metabolic control; core variables (i.e., glucose and lipid metabolic disorders, peripheral inflammation) were also not adjusted to avoid over-adjustment.

P<0.05 indicated statistical significance (19), with analyses performed using SPSS 27.0.

COX regression was not used in the cohort study due to uniform 9-month follow-up; onset time of POCPS-CCCS was not a focus, and this design aligned with fixed follow-up exposure-outcome analysis requirements.

GPower 3.1.9.7 software validated sample size adequacy, confirming sufficient statistical power for detecting target associations in both study components.

### 2.6 Ethics requirements

This study was approved by the Medical Ethics Committee of the First Affiliated Hospital of Xinxiang Medical University (Approval No.: EC025586) and conducted in accordance with the World Medical Association Declaration of Helsinki.

## 3 Results

### 3.1 Participants

At baseline, 610 patients meeting the inclusion criteria were initially assigned to the chronic pain group (n=197) or control group (n=413). During the follow-up, 97 patients experienced tumor recurrence (36 in the chronic pain group [18.3%] and 61 in the control group [14.5%]), with no significant difference in recurrence rates between groups (P = 0.268). These patients with recurrence were excluded from further analysis. No losses to follow-up, refusals, or deaths occurred during the study period.

Ultimately, the case-control study included 161 patients in the chronic pain group and 352 in the control group. For the cohort study, these 352 control patients were further subdivided into the metabolic disorder subgroup (n=126) and metabolic normal subgroup (n=226).

### 3.2 Case-control study

In **Table 1**, the chronic pain group showed a higher proportion of essential hypertension (P = 0.048), cardiovascular diseases (P = 0.016), and cerebrovascular diseases (P = 0.026) compared with the control group. No statistically significant differences were observed in demographic characteristics, tumor pathological data, or postoperative treatment measures between the groups (all P>0.05).

**Table 1:** Characteristics of patients in the case-control study.

Variable	Chronic pain group	Control group	T/χ² value	P value
Total (n)	161 (100.0)	352 (100.0)	−	−
Demographic data				
Age (year)	62.9 ± 7.5	62.4 ± 7.4	0.721	0.471
Male (n)	91 (56.5)	180 (51.1)	1.286	0.257
Han nationality (n)	148 (91.9)	313 (88.9)	1.095	0.295
History of disease				
Essential hypertension (n)	105 (65.2)	197 (56.0)	3.905	0.048
Cardiovascular diseases (n)	78 (48.4)	131 (37.2)	5.772	0.016
Cerebrovascular diseases (n)	35 (21.7)	49 (13.9)	4.932	0.026
Tumor marker (At baseline)				
CEA (μg/l)	3.0 ± 1.7	2.8 ± 1.7	1.153	0.249
CA199 (U/ml)	20.8 ± 11.7	19.0 ± 10.8	1.702	0.089
CA242 (U/ml)	11.9 ± 7.0	11.2 ± 6.3	0.996	0.320
Tumor site				
Rectum (n)	93 (57.8)	183 (52.0)	1.482	0.223
Colon (n)	68 (42.2)	169 (48.0)		
Tumor size				
Greater than 5 cm (n)	86 (53.4)	166 (47.2)	1.731	0.188
Less than 5 cm (n)	75 (46.6)	186 (52.8)		
Histological type				
Adenocarcinoma (n)	147 (91.3)	335 (95.2)	2.908	0.088
Other (n)	14 (8.7)	17 (4.8)		
Differentiation grade				
High to moderate (n)	104 (64.6)	250 (71.0)	2.133	0.144
Low to undifferentiated (n)	57 (35.4)	102 (29.0)		
Tumor stage				
Stage I - II (n)	95 (59.0)	229 (65.1)	1.738	0.187
Stage III (n)	66 (41.0)	123 (34.9)		
Postoperative treatment				
Adjuvant chemotherapy (n)	89 (55.3)	177 (50.3)	1.104	0.293
Targeted therapy (n)	20 (12.4)	32 (9.1)	1.346	0.246
Immunotherapy (n)	17 (10.6)	24 (6.8)	2.102	0.147
TCM therapy (n)	41 (25.5)	73 (20.7)	1.428	0.232

CEA, Carcinoembryonic antigen; CA199, Cancer antigen 199; CA242, Cancer antigen 242; TCM, Traditional Chinese medicine. Continuous variables are expressed as mean ± standard deviation, and categorical variables are expressed in terms of frequencies and proportions. A P-value less than 0.05 indicates that the difference is statistically significant.

In **Table 2**, the chronic pain group had a higher prevalence of glucose metabolic disorders (P = 0.003) and lipid metabolic disorders (P = 0.001), as well as a higher proportion of patients receiving hypoglycemic (P = 0.011) and antilipemic treatments (P = 0.040), compared with the control group. Additionally, the chronic pain group exhibited higher levels of peripheral IL-1β (P<0.001), IL-6 (P<0.001), and TNF-α (P<0.001), along with higher anxiety scores (P<0.001), higher depression scores (P<0.001), and lower quality of life scores (P<0.001).

**Table 2:** Glucose and lipid metabolic disorders, peripheral inflammation, POCPS-CCCS, psychological status, and quality of life in the case-control study.

Variable (at baseline)	Chronic pain group	Control group	T/χ² value	P value
Total (n)	161 (100.0)	352 (100.0)	−	−
Glucose metabolic disorders				
IFG (n)	29 (18.0)	42 (11.9)	3.425	0.064
IGT (n)	35 (21.7)	52 (14.8)	3.807	0.051
T2DM (n)	27 (16.8)	32 (9.1)	6.401	0.011
Total (n)	54 (33.5)	75 (21.3)	8.784	0.003
Lipid metabolic disorders				
HTG (n)	41 (25.5)	62 (17.6)	4.245	0.039
HC (n)	36 (22.4)	56 (15.9)	3.124	0.077
HLDLC (n)	32 (19.9)	38 (10.8)	7.730	0.005
Total (n)	61 (37.9)	83 (23.6)	11.202	0.001
Medication history				
Hypoglycemic treatment (n)	27 (16.8)	32 (9.1)	6.401	0.011
Antilipemic treatment (n)	34 (21.1)	49 (13.9)	4.220	0.040
Peripheral inflammation				
IL - 1β (pg/mL)	17.68 ± 4.33	12.73 ± 4.54	11.634	<0.001
IL - 6 (pg/mL)	11.91 ± 3.41	8.94 ± 3.46	9.054	<0.001
TNF - α (pg/mL)	16.73 ± 4.33	12.21 ± 4.78	10.244	<0.001
POCPS-CCCS				
Present (n)	161 (100.0)	−	−	−
Absent (n)	−	352 (100.0)		
Psychological status				
HADS (anxiety) score	11.2 ± 2.5	8.2 ± 3.1	10.936	<0.001
HADS (depression) score	10.5 ± 2.3	7.7 ± 2.9	10.928	<0.001
Quality of life				
FLIC score	75.7 ± 4.1	82.8 ± 5.8	14.073	<0.001

IFG, Impaired fasting glucose; IGT, Impaired glucose tolerance; T2DM, Type 2 diabetes mellitus; HTG, Hypertriglyceridemia; HC, Hypercholesterolemia; HLDLC, High LDL - cholesterol; IL - 1β, Interleukin - 1β; IL - 6, Interleukin - 6; TNF - α, Tumor necrosis factor - α; POCPS-CCCS, Post-operative chronic pain syndrome after colorectal cancer surgery; HADS, Hospital anxiety and depression scale; FLIC, Functional living index-cancer. Continuous variables are expressed as mean ± standard deviation, and categorical variables are expressed in terms of frequencies and proportions. A P-value less than 0.05 indicates that the difference is statistically significant.

**Figure 2:**
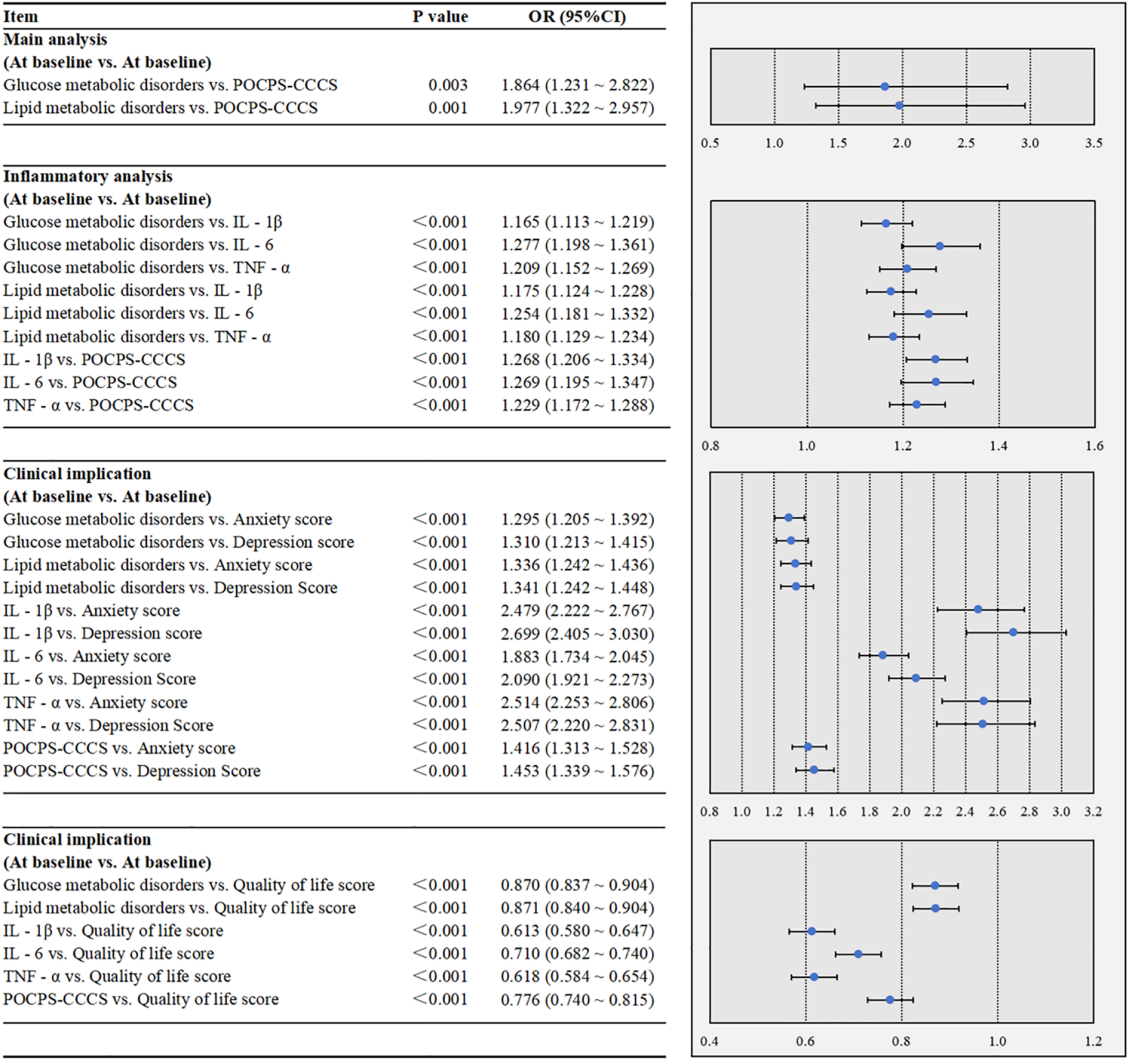
Multivariate associations among glucose and lipid metabolic disorders, peripheral inflammation, POCPS-CCCS, psychological status, and quality of life in the case-control study. POCPS-CCCS, Post-operative chronic pain syndrome after colorectal cancer surgery; IL - 1β, Interleukin - 1β; IL - 6, Interleukin - 6; TNF - α, Tumor Necrosis Factor - α. A P-value less than 0.05 indicates that the association is statistically significant. In the forest plots, the dots represent ORs, and the horizontal bars represent 95% CIs.

In **Figure 2**, the multivariate analysis revealed that glucose metabolic disorders (P = 0.003) and lipid metabolic disorders (P = 0.001) were each significantly associated with an increased risk of POCPS-CCCS. The inflammatory analysis showed that both glucose and lipid metabolic disorders were significantly associated with elevated peripheral IL-1β (both P<0.001), IL-6 (both P<0.001), and TNF-α (both P<0.001). Moreover, elevated levels of IL-1β (P<0.001), IL-6 (P<0.001), and TNF-α (P<0.001) were each significantly associated with an increased risk of POCPS-CCCS. The clinical implication analysis demonstrated that glucose metabolic disorders, lipid metabolic disorders, elevated peripheral IL-1β/IL-6/TNF-α, and POCPS-CCCS were all significantly associated with higher anxiety scores (all P<0.001), higher depression scores (all P<0.001), and lower quality of life scores (all P<0.001).

### 3.3 Cohort study

In **Table 3**, the metabolic disorder subgroup had a higher proportion of essential hypertension (P = 0.001), cardiovascular diseases (P = 0.001), and cerebrovascular diseases (P<0.001) compared with the metabolic normal subgroup. No significant differences were observed in other research variables between the subgroups (all P>0.05).

**Table 3:** Characteristics of patients in the cohort study.

Variable	Metabolic disorder subgroup	Metabolic normal subgroup	T/χ² value	P value
Total (n)	126 (100.0)	226 (100.0)	−	−
Demographic data				
Age (year)	62.3 ± 7.3	62.4 ± 7.4	0.079	0.937
Male (n)	65 (51.6)	115 (50.9)	0.016	0.899
Han nationality (n)	116 (92.1)	197 (87.2)	1.968	0.161
History of disease				
Essential hypertension (n)	85 (67.5)	112 (49.6)	10.521	0.001
Cardiovascular diseases (n)	62 (49.2)	69 (30.5)	12.075	0.001
Cerebrovascular diseases (n)	30 (23.8)	19 (8.4)	16.016	<0.001
Tumor marker (At baseline)				
CEA (μg/l)	2.95 ± 1.69	2.76 ± 1.70	0.986	0.325
CA199 (U/ml)	19.89 ± 10.13	18.44 ± 11.14	1.202	0.230
CA242 (U/ml)	11.64 ± 6.34	11.02 ± 6.22	0.891	0.374
Tumor site				
Rectum (n)	72 (57.1)	111 (49.1)	2.089	0.149
Colon (n)	54 (42.9)	115 (50.9)		
Tumor size				
Greater than 5 cm (n)	65 (51.6)	101 (44.7)	1.544	0.214
Less than 5 cm (n)	61 (48.4)	125 (55.3)		
Histological type				
Adenocarcinoma (n)	118 (93.7)	217 (96.0)	0.986	0.321
Other (n)	8 (6.3)	9 (4.0)		
Differentiation grade				
High to moderate (n)	84 (66.7)	166 (73.5)	1.809	0.179
Low to undifferentiated (n)	42 (33.3)	60 (26.5)		
Tumor stage				
Stage I - II (n)	76 (60.3)	153 (67.7)	1.939	0.164
Stage III (n)	50 (39.7)	73 (32.3)		
Postoperative treatment				
Adjuvant chemotherapy (n)	70 (55.6)	107 (47.3)	2.181	0.140
Targeted therapy (n)	14 (11.1)	18 (8.0)	0.969	0.325
Immunotherapy (n)	11 (8.7)	13 (5.8)	1.129	0.288
TCM therapy (n)	29 (23.0)	44 (19.5)	0.619	0.431

CEA, Carcinoembryonic antigen; CA199, Cancer antigen 199; CA242, Cancer antigen 242; TCM, Traditional Chinese medicine. Continuous variables are expressed as mean ± standard deviation, and categorical variables are expressed in terms of frequencies and proportions. A P-value less than 0.05 indicates that the difference is statistically significant.

In **Table 4**, the metabolic disorder subgroup exhibited significantly higher baseline levels of IL-1β (P<0.001), IL-6 (P<0.001), and TNF-α (P<0.001) compared with the metabolic normal subgroup. During the follow-up, new-onset POCPS-CCCS was more common in the metabolic disorder subgroup (P<0.001). At the follow-up end, the metabolic disorder subgroup had higher anxiety (P<0.001) and depression scores (P<0.001), and lower quality of life scores (P<0.001).

**Table 4:** Glucose and lipid metabolic disorders, peripheral inflammation, POCPS-CCCS, psychological status, and quality of life in the cohort study.

Variable	Metabolic disorder subgroup	Metabolic normal subgroup	T/χ² value	P value
Total (n)	126 (100.0)	226 (100.0)	−	−
At baseline				
Glucose metabolic disorders				
IFG (n)	42 (33.3)	−	−	−
IGT (n)	52 (41.3)	−	−	−
T2DM (n)	32 (25.4)	−	−	−
Total (n)	75 (59.5)	−	−	−
Lipid metabolic disorders				
HTG (n)	62 (49.2)	−	−	−
HC (n)	56 (44.4)	−	−	−
HLDLC (n)	38 (30.2)	−	−	−
Total (n)	83 (65.9)	−	−	−
Medication history				
Hypoglycemic treatment (n)	32 (25.4)	−	−	−
Antilipemic treatment (n)	49 (38.9)	−	−	−
Peripheral inflammation				
IL - 1β (pg/mL)	14.97 ± 4.01	11.47 ± 4.34	7.460	<0.001
IL - 6 (pg/mL)	10.98 ± 3.04	7.80 ± 3.16	9.163	<0.001
TNF - α (pg/mL)	15.03 ± 4.13	10.63 ± 4.38	9.202	<0.001
During the follow-up				
POCPS-CCCS				
Absence (n)	67 (53.2)	162 (71.7)	12.188	<0.001
Present (n)	59 (46.8)	64 (28.3)		
At the follow-up end				
Psychological status				
HADS (anxiety) score	12.3 ± 3.3	9.8 ± 3.3	6.826	<0.001
HADS (depression) score	12.2 ± 3.2	9.6 ± 3.1	7.463	<0.001
Quality of life				
FLIC score	71.7 ± 6.6	76.6 ± 6.1	7.063	<0.001

IFG, Impaired Fasting Glucose; IGT, Impaired Glucose Tolerance; T2DM, Type 2 Diabetes Mellitus; HTG, Hypertriglyceridemia; HC, Hypercholesterolemia; HLDLC, High LDL - Cholesterol; IL - 1β, Interleukin - 1β; IL - 6, Interleukin - 6; TNF - α, Tumor Necrosis Factor - α; POCPS-CCCS, Post-operative chronic pain syndrome after colorectal cancer surgery; HADS, Hospital anxiety and depression scale; FLIC, Functional living index-cancer. Metabolic normal subgroup had no glucose and lipid metabolic disorders at baseline, so relevant data are marked as “−”.

Continuous variables are expressed as mean ± standard deviation, and categorical variables are expressed in terms of frequencies and proportions. A P-value less than 0.05 indicates that the difference is statistically significant.

**Figure 3:**
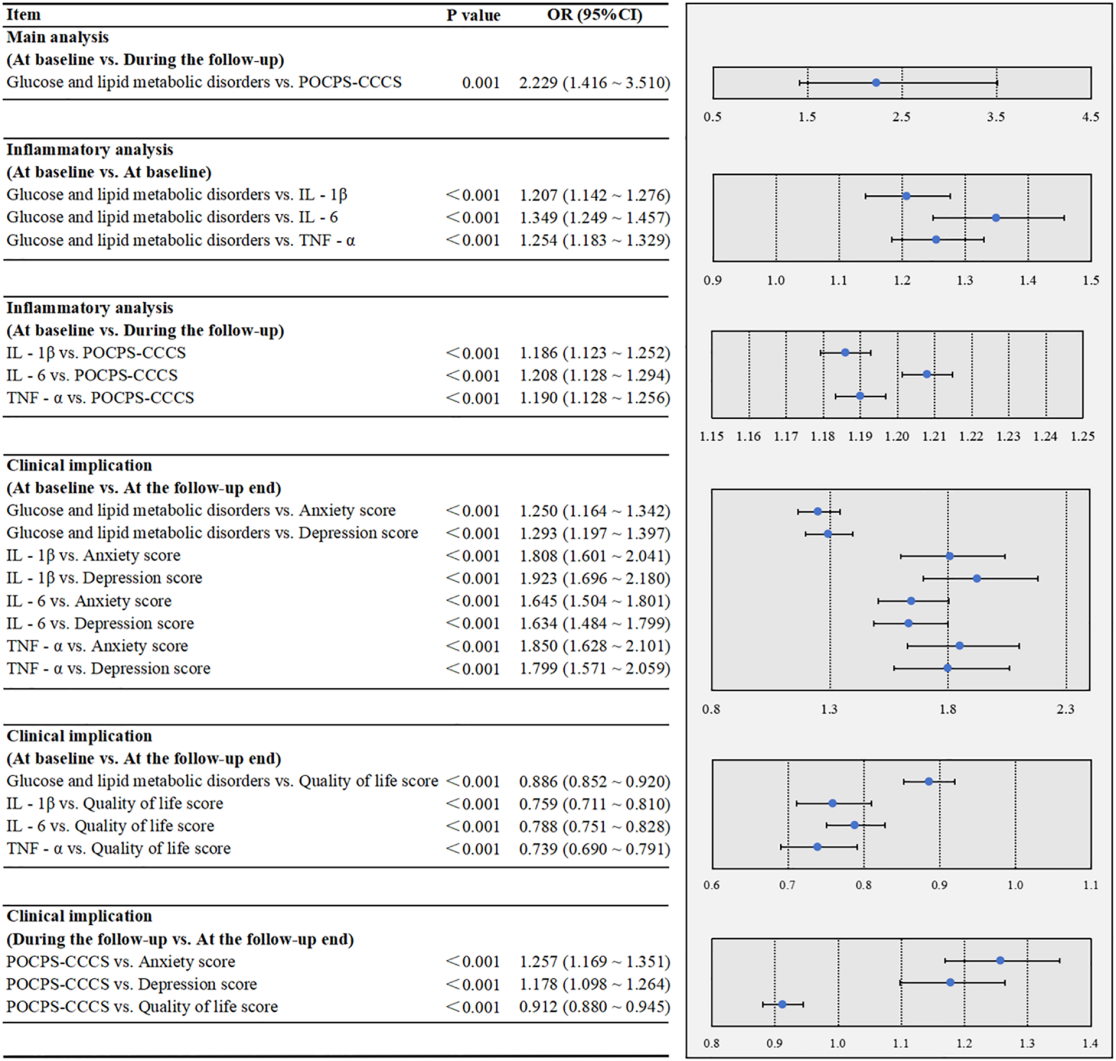
Multivariate associations among glucose/lipid metabolic disorders, peripheral inflammation, POCPS-CCCS, psychological status, and quality of life in the cohort study. POCPS-CCCS, Post-operative chronic pain syndrome after colorectal cancer surgery; IL - 1β, Interleukin - 1β; IL - 6, Interleukin - 6; TNF - α, Tumor Necrosis Factor - α. A P-value less than 0.05 indicates that the association is statistically significant. In the forest plots, the dots represent ORs, and the horizontal bars represent 95% CIs.

In **Figure 3**, the multivariate analysis indicated that glucose and lipid metabolic disorders at baseline were associated with an increased risk of POCPS-CCCS during the follow-up (P = 0.001). The inflammatory analysis showed that glucose and lipid metabolic disorders were significantly associated with elevated baseline IL-1β (P<0.001), IL-6 (P<0.001), and TNF-α (P<0.001) at baseline. Elevated levels of these inflammatory factors at baseline were also significantly associated with an increased risk of POCPS-CCCS during the follow-up (all P<0.001). The clinical implication analysis revealed that glucose and lipid metabolic disorders and elevated these inflammatory factors at baseline were significantly associated with higher depression (all P<0.001) and anxiety scores (all P<0.001) and lower quality of life scores (all P<0.001) at the follow-up end. Additionally, POCPS-CCCS during the follow-up was significantly associated with higher depression (P<0.001) and anxiety scores (P<0.001) and lower quality of life scores (P<0.001) at the follow-up end.

### 3.4 Statistical power validation

For the case-control study, based on an OR value of 1.864 for the association between glucose metabolic disorders and POCPS-CCCS, the estimated effect size was 0.25. With a significance level of 0.05 and statistical power of 0.95, the minimum required sample size was 164 cases. The actual sample size (513 cases) far exceeded this threshold.

For the cohort study, using an OR value of 2.229 for the association between glucose and lipid metabolic disorders and POCPS-CCCS, the estimated effect size was 0.30. With the same significance level (0.05) and statistical power (0.95), the minimum required sample size was 111 cases. The actual sample size (352 cases) also exceeded this requirement.

These results confirmed sufficient statistical power for both study components.

## 4 Discussion

At present, the pathogenesis and influencing factors of POCPS-CCCS remain underrecognized, yet its impact on patients’ postoperative quality of life is substantial. This study employed a combined case-control and cohort design to evaluate the roles of metabolic disorders and peripheral inflammation in POCPS-CCCS development and their subsequent clinical implications.

Our findings confirm that glucose and lipid metabolic disorders are associated with an increased risk of POCPS-CCCS. Notably, these conclusions are derived from the integration of cross-sectional (case-control) and longitudinal (cohort) results, which mitigates the risk of reverse causation. To address potential confounding, the multivariate logistic or linear regression adjusted for imbalances in factors such as comorbidities, confirming the independence of the observed associations. Additionally, rigorous monitoring and exclusion of patients with tumor recurrence throughout the study — a critical confounder in POCPS-CCCS research — ensured the reliability of our results.

Currently, studies using multiple disease models have mechanistically confirmed a close link between glucose and lipid metabolic disorders and chronic inflammation (20, 21). Inflammatory mechanisms also play roles in nerve injury repair and pain perception regulation (22, 23). While these findings do not target the mechanisms of POCPS-CCCS, they still offer valuable references for investigating this condition’s pathogenesis and developing interventions. In line with this, our study found that elevated levels of peripheral inflammatory factors (IL-1β, IL-6, TNF-α) were associated with the two metabolic disorders and an increased risk of POCPS-CCCS. These findings not only confirm the possible role of peripheral inflammation in POCPS-CCCS pathogenesis, but also suggest that the metabolic disorders and inflammation may potentially act synergistically to drive the development of this postoperative complication.

The most significant consequence of POCPS-CCCS lies not in direct effects on colorectal cancer prognosis, but in its impact on patients’ quality of life: persistent pain increases the risk of anxiety and depression, erodes treatment adherence, and indirectly affects long-term outcomes (24, 25). Our results extend this understanding by showing that metabolic and inflammatory disorders — alongside POCPS-CCCS itself — are significantly associated with worse psychological status and lower quality of life. This seemingly suggests the clinical value of targeting metabolic and inflammatory disorders to reduce the risk of POCPS-CCCS and improve patient well-being.

Surgical technique is a potential confounding factor in POCPS-CCCS research, as varying degrees of tissue and nerve trauma may influence chronic pain incidence. In our study, all patients underwent conventional colorectal cancer resection procedures (e.g., right hemicolectomy, anterior rectal resection) (26), with no significant differences in surgical type distribution across study groups (results not shown). This ensures that our findings are not biased by variations in surgical approaches.

The primary strength of this study is its dual design, which allows cross-sectional and longitudinal results to validate each other, enhancing the robustness of our conclusions. However, a key design consideration was the separate analysis of glucose and lipid metabolic disorders in the case-control study, contrasted with their combined analysis in the cohort study. This choice was driven by the smaller sample size in the cohort study, where combined analysis maximized statistical power. Given the interconnected nature of glucose and lipid metabolism — shared regulatory mechanisms and high clinical co-occurrence (e.g., diabetes with hyperlipidemia) — this approach is clinically relevant. However, the inability to assess the distinct roles of glucose and lipid metabolic disorders in the cohort study remains a limitation. Future research with larger samples should clarify their individual contributions and explore underlying mechanisms in depth.

Several other limitations of this study should also be further addressed. First, although we controlled for as many confounding factors as possible using multivariate logistic or linear regression, residual confounding may still exist — particularly the individual differences in postoperative pain management protocols, which were not separately included in the analysis and may have exerted potential impacts on the results. Second, all study data were derived from a single center, and the lack of external validation and multicenter data support limits the generalizability of our findings. Third, this study did not conduct mechanistic investigations, such as detecting biomarkers related to nerve injury or assessing neuropathological changes via imaging; additionally, variables that may influence the development of chronic pain—including baseline pain sensitivity and psychosocial factors (e.g., preoperative anxiety, pain catastrophizing) — were not incorporated. Finally, due to constraints related to funding and patient compliance, we were unable to validate mechanisms through animal models or interventional studies. Moreover, the study focused solely on glucose and lipid metabolism without involving other metabolic pathways. All these limitations should be addressed in future studies with larger sample sizes and multicenter designs, combined with mechanistic exploration, to deepen our understanding of the pathogenesis of POCPS-CCCS.

In conclusion, our findings preliminarily suggest that glucose and lipid metabolic disorders, alongside peripheral inflammation, may be involved in promoting the development of POCPS-CCCS. These metabolic, inflammatory, and pain-related abnormalities might collectively impact patients’ physical and mental health. Improving metabolic control and reducing inflammation could thus represent a potentially promising strategy to help lower POCPS-CCCS risk, support health maintenance, and may contribute to improving long-term prognosis in colorectal cancer survivors.

## Data Availability

The raw data supporting the conclusions of this article will be made available by the authors, without undue reservation.

## References

[1] Alharbi RA Elfeki H Emmertsen KJ Mortensen AR Drewes AM Christensen P. Chronic pain after colon cancer surgery: Translation and validation of a scoring system. Colorectal Dis. (2023) 25:202–10. doi: 10.1111/codi.16339, PMID: 36100354

[2] Stamer UM Lavand’homme P Hofer DM Barke A Korwisi B. Chronic postsurgical pain in the ICD-11: implications for anaesthesiology and pain medicine. Br J Anaesth. (2025) S0007-0912(25)00094-7. doi: 10.1016/j.bja.2025.02.005, PMID: 40089399

[3] Jin J Chen Q Min S Du X Zhang D Qin P. Prevalence and predictors of chronic postsurgical pain after colorectal surgery: A prospective study. Colorectal Dis. (2021) 23:1878–89. doi: 10.1111/codi.15640, PMID: 33738887

[4] Rosendahl A Barsøe IM Ott V Brandstrup B Thomsen T Møller AM. Chronic postsurgical pain following gastrointestinal surgery -A scoping review. Acta Anaesthesiol Scand. (2025) 69:e14560. doi: 10.1111/aas.14560, PMID: 39611389

[5] Jin J Zhang T Xiong X Chen H Jiang Y He S. A prospective study of chronic postsurgical pain in elderly patients: incidence, characteristics and risk factors. BMC Geriatr. (2023) 23:289. doi: 10.1186/s12877-023-04006-w, PMID: 37173634 PMC10182592

[6] Habibi BA Kim C Elsharkawy H. Persistent and chronic perioperative pain after cancer surgery. Curr Oncol Rep. (2022) 24:215–22. doi: 10.1007/s11912-021-01152-5, PMID: 35061194

[7] Li Y Kong E Ding R Chu R Lu J Deng M. Hyperglycemia-induced Sirt3 downregulation increases microglial aerobic glycolysis and inflammation in diabetic neuropathic pain pathogenesis. CNS Neurosci Ther. (2024) 30:e14913. doi: 10.1111/cns.14913, PMID: 39123294 PMC11315676

[8] Osmanlıoğlu HÖ Nazıroğlu M. Resveratrol modulates diabetes-induced neuropathic pain, apoptosis, and oxidative neurotoxicity in mice through TRPV4 channel inhibition. Mol Neurobiol. (2024) 61:7269–86. doi: 10.1007/s12035-024-04311-4, PMID: 38976129 PMC11339089

[9] Zammit A Coquet J Hah J El Hajouji O Asch SM Carroll I. Postoperative opioid prescribing patients with diabetes: Opportunities for personalized pain management. PloS One. (2023) 18:e0287697. doi: 10.1371/journal.pone.0287697, PMID: 37616195 PMC10449216

[10] Fu CN Wei H Gao WS Song SS Yue SW Qu YJ. Obesity increases neuropathic pain via the AMPK-ERK-NOX4 pathway in rats. Aging (Albany NY). (2021) 13:18606–19. doi: 10.18632/aging.203305, PMID: 34326272 PMC8351691

[11] Llombart-Blanco R Mariscal G Barrios C de la Rubia Ortí JE Llombart-Ais R. Effects of obesity on function, pain, and complications after rotator cuff repair: An updated systematic review and meta-analysis. Obes Res Clin Pract. (2025) 19:193–201. doi: 10.1016/j.orcp.2025.04.010, PMID: 40300915

[12] Wedel S Hahnefeld L Schreiber Y Namendorf C Heymann T Uhr M. SAFit2 ameliorates paclitaxel-induced neuropathic pain by reducing spinal gliosis and elevating pro-resolving lipid mediators. J Neuroinflamm. (2023) 20:149. doi: 10.1186/s12974-023-02835-5, PMID: 37355700 PMC10290418

[13] Dariya B Aliya S Merchant N Alam A Nagaraju GP. Colorectal cancer biology, diagnosis, and therapeutic approaches. Crit Rev Oncog. (2020) 25:71–94. doi: 10.1615/CritRevOncog.2020035067, PMID: 33389859

[14] Rahman S Kidwai A Rakhamimova E Elias M Caldwell W Bergese SD. Clinical diagnosis and treatment of chronic pain. Diagnostics (Basel). (2023) 13:3689. doi: 10.3390/diagnostics13243689, PMID: 38132273 PMC10743062

[15] Kuo FY Cheng KC Li Y Cheng JT. Oral glucose tolerance test in diabetes, the old method revisited. World J Diabetes. (2021) 12:786–93. doi: 10.4239/wjd.v12.i6.786, PMID: 34168728 PMC8192259

[16] Shah K Maghsoudlou P. Enzyme-linked immunosorbent assay (ELISA): the basics. Br J Hosp Med (Lond). (2016) 77:C98–101. doi: 10.12968/hmed.2016.77.7.C98, PMID: 27388394

[17] Bjelland I Dahl AA Haug TT Neckelmann D. The validity of the Hospital Anxiety and Depression Scale. An updated literature review. . J Psychosom Res. (2002) 52:69–77. doi: 10.1016/S0022-3999(01)00296-3, PMID: 11832252

[18] Fong DY Lee AH Tung SY Wong JY Chan YM Goh CR. The Functional Living Index-Cancer is a reliable and valid instrument in Chinese cancer patients. Qual Life Res. (2014) 23:311–6. doi: 10.1007/s11136-013-0456-z, PMID: 23775604

[19] Yan F Robert M Li Y. Statistical methods and common problems in medical or biomedical science research. Int J Physiol Pathophysiol Pharmacol. (2017) 9:157–63. PMC5698693 29209453

[20] Poznyak A Grechko AV Poggio P Myasoedova VA Alfieri V Orekhov AN. The diabetes mellitus-atherosclerosis connection: the role of lipid and glucose metabolism and chronic inflammation. Int J Mol Sci. (2020) 21:1835. doi: 10.3390/ijms21051835, PMID: 32155866 PMC7084712

[21] Mitchelson KAJ O’Connell F O’Sullivan J Roche HM. Obesity, dietary fats, and gastrointestinal cancer risk-potential mechanisms relating to lipid metabolism and inflammation. Metabolites. (2024) 14:42. doi: 10.3390/metabo14010042, PMID: 38248845 PMC10821017

[22] Svačina MKR Gao T Sprenger-Svačina A Lin J Ganesh BP Lee J. Rejuvenating fecal microbiota transplant enhances peripheral nerve repair in aged mice by modulating endoneurial inflammation. Exp Neurol. (2024) 376:114774. doi: 10.1016/j.expneurol.2024.114774, PMID: 38599367

[23] Dworsky-Fried Z Faig CA Vogel HA Kerr BJ Taylor AMW. Central amygdala inflammation drives pain hypersensitivity and attenuates morphine analgesia in experimental autoimmune encephalomyelitis. Pain. (2022) 163:e49–61. doi: 10.1097/j.pain.0000000000002307, PMID: 33863858

[24] Poço Gonçalves J Veiga D Araújo A. Chronic pain, functionality and quality of life in cancer survivors. Br J Pain. (2021) 15:401–10. doi: 10.1177/2049463720972730, PMID: 34840788 PMC8611292

[25] Cox-Martin E Anderson-Mellies A Borges V Bradley C. Chronic pain, health-related quality of life, and employment in working-age cancer survivors. J Cancer Surviv. (2020) 14:179–87. doi: 10.1007/s11764-019-00843-0, PMID: 31828603 PMC7473420

[26] Rentsch M Schiergens T Khandoga A Werner J. Surgery for colorectal cancer - trends, developments, and future perspectives. Visc Med. (2016) 32:184–91. doi: 10.1159/000446490, PMID: 27493946 PMC4945784

